# Breaking the Cycle of Malnutrition: The Role of Food and Nutrition Literacy in Addressing Food Insecurity Among Lebanese Adolescents

**DOI:** 10.3390/nu17193140

**Published:** 2025-09-30

**Authors:** Elie Ghadban, Tigresse Boutros, Souheil Hallit, Nikolaos Tzenios, Yonna Sacre, Maha Hoteit

**Affiliations:** 1 Department of Plastic Surgery, Saint George University of Beirut Achrafieh, Beirut, Lebanon; ghadban.e@sgub.edu.lb; 2 School of Medicine and Medical Sciences, Holy Spirit University of Kaslik, Jounieh P.O. Box 446, Lebanon; tigresseboutros@gmail.com (T.B.); souheilhallit@hotmail.com (S.H.); 3 Department of Psychology, College of Humanities, Effat University, Jeddah 21478, Saudi Arabia; 4 Applied Science Research Center, Applied Science Private University, Amman 11937, Jordan; 5 Faculty of Public Health, Charisma University, London EC1V 7QE, UK; 6 Department of Nutrition and Food Sciences, Faculty of Arts and Sciences, Holy Spirit University of Kaslik (USEK), Jounieh P.O. Box 446, Lebanon; yonnasacre@usek.edu.lb; 7 PHENOL Research Program, Faculty of Public Health, Section 1, Lebanese University, Beirut P.O. Box 6573, Lebanon; 8 Department of Primary Care and Population Health, University of Nicosia Medical School, 2417 Nicosia, Cyprus; 9 INSPECT-LB (Institut National de Santé Publique, d’Épidémiologie Clinique et de Toxicologie-Liban), Beirut, Lebanon

**Keywords:** food insecurity, nutrition literacy, food literacy, adolescents, stunting, obesity/overweight, Lebanon

## Abstract

**Background:** Undernutrition and overnutrition are considered a rising challenge among adolescents in low- and middle-income countries, including Lebanon, where overlapping economic, political, and public health crises have worsened food insecurity. Food and nutrition literacy in adolescents may serve as protective factors against food insecurity and its nutritional consequences. This study aims to evaluate the associations between adolescent and parental food and nutrition literacy with household and adolescent food insecurity, and explores their relationship with stunting and overweight/obesity. **Methodology:** A cross-sectional survey was conducted between March and July 2022 among 442 Lebanese adolescents (10–18 years) and one parent/caregiver per household, recruited via snowball sampling from all eight governorates. Validated tools assessed adolescent food and nutrition literacy, parental food literacy, household/adolescent food insecurity, and anthropometric status. Chi-square, *t*-tests, and multivariable logistic regressions identified factors associated with food insecurity, stunting, and overweight/obesity. **Results:** Higher adolescent food and nutrition literacy was significantly associated with lower odds of severe food insecurity (aOR = 0.43, 95% CI: 0.26–0.70). Higher parental food literacy scores were linked to reduced odds of severe household food insecurity (aOR = 0.94, 95% CI: 0.90–0.98). Severe food insecurity was more likely in households in Akkar and among adolescents not attending school or with poor food and nutrition literacy. Overweight/obesity was positively associated with attending private school and higher parental body mass index, but inversely associated with higher child food security and household crowding index. No significant association was found between food insecurity and stunting. **Conclusions:** Both adolescent and parental food and nutrition literacy are protective against severe food insecurity, highlighting the value of literacy-focused interventions alongside economic support measures. Addressing both educational and structural determinants may help break the cycle of malnutrition in crisis-affected Lebanese youth.

## 1. Introduction

In the trajectory of human development, adolescence represents the second and last call for catching up on growth opportunities missed in childhood. Yet, for many young people, the conditions shaping physical development are already set by the enduring effects of early-life deprivation and the household environment in which they live [1,2]. In this context, both individual and family-level factors play pivotal roles in determining nutrition outcomes [3,4]. While adolescents gain more autonomy over their food choices during this stage, parents remain central in shaping the home food environment through purchasing decisions, food preparation practices, and nutrition modeling [3,4].

Malnutrition in adolescence, whether undernutrition manifesting as stunting or overnutrition leading to overweight and obesity, remains a critical global public health challenge, particularly in low- and middle-income countries (LMICs) undergoing nutritional transitions [1,5]. According the World Health Organization (WHO), stunting is defined as a height-for-age of more than two standard deviations below the median of the WHO Child Growth Standards [6], while overweight and obesity are characterized by excessive fat accumulation that may impair health, often assessed using age- and sex-specific body mass index (BMI)-for-age cutoffs [7]. Both forms of malnutrition have profound and lasting effects: stunting is linked to impaired cognitive development, lower academic performance, reduced economic productivity, and increased risk of chronic diseases in adulthood [8], whereas overweight and obesity are associated with early onset of non-communicable diseases, psychosocial difficulties, and diminished quality of life [9].

Despite global initiatives aimed at improving adolescent nutrition, the “double burden” of malnutrition, referring to the coexistence of undernutrition (including stunting) alongside overweight and obesity within the same populations, households, and even individuals, remains a pressing issue, particularly in LMICs undergoing rapid nutritional transitions [10]. In the Middle East and North Africa (MENA) region, both stunting and adolescent overweight persist as significant public health challenges despite improvements in healthcare access [1,11]. Globally, data from UNICEF emphasize the magnitude of the problem, with food deprivation and acute hunger affecting nearly 25.9% of the global population, or around 67 million people worldwide [12,13]. 21.3% of children under five are stunted, while in the MENA region, prevalence reaches about 24% [1]. Particularly in Lebanon, the persistence of both stunting and adolescent overweight reflects the impact of socioeconomic crises and dietary transitions on youth nutrition [14,15].

### 1.1. Food and Nutrition Literacy in Adolescence and Households

Against this backdrop, attention has increasingly shifted toward the role of food and nutrition literacy as an underlying determinant of dietary behaviors and nutrition outcomes within households [14,16]. Food literacy (FL) is described as the set of interconnected knowledge, skills, and behaviors necessary to manage, select, prepare, and consume foods in ways that meet nutritional needs and guide food intake [14]. Nutrition literacy (NL), on the other hand, refers to the capacity to access, interpret, and comprehend fundamental nutrition information for the purpose of making informed dietary choices [17]. Increasingly, FL and NL are seen as two dimensions of a unified construct, food and nutrition literacy, because of their interdependence in promoting healthy dietary behaviors and reducing vulnerability to malnutrition [18].

Beyond influencing food choices, food and nutrition literacy constitutes a key determinant of food security, particularly in the utilization pillar, which encompasses the safe storage, preparation, and consumption of food [19,20]. This dimension is critical for adolescents, who are gradually acquiring greater autonomy in food-related decisions, as well as for parents, who continue to shape the home food environment through purchasing patterns, meal preparation, and nutrition modeling [21]. Insufficient food and nutrition literacy, whether at the adolescent or parental level, may limit the effective use of available foods, leading to compromised diet quality even when physical access is adequate [19,22,23].

### 1.2. Food Insecurity, the Double Burden of Malnutrition, and the Role of Food and Nutrition Literacy

Among the many drivers of adolescent malnutrition, food insecurity (FI) stands out as both a direct and indirect determinant [24]. FI refers to the lack of reliable access to sufficient, safe, and nutritious food, often resulting in disrupted eating patterns or reduced food intake due to limited financial or other essential resources [25]. Its impact extends to growth, dietary diversity, mental health, academic performance, and overall quality of life [26]. Beyond nutritional inadequacies, food insecurity often compels households to adopt coping strategies such as reducing meal frequency, compromising dietary quality by relying on inexpensive nutrient-poor foods, and experiencing increased psychosocial stress, which collectively can negatively affect adolescent growth and development [27]. Particularly in Lebanon, FI has reached concerning prevalence, with more than half of households with children reporting some degree of insecurity, and severe forms increasingly concentrated among vulnerable groups [14,15]. A recent study by Hoteit et al. examined how Lebanese adolescents are an affected subcategory, facing a convergence of nutritional burdens in which 6.7% are stunted, 4.7% are thin, 19.3% are overweight, 12.9% are obese, and 16.7% are anemic, while more than 40% live in households consuming undiversified diets [28].

Studies have shown that FI is associated with both undernutrition, through nutrient deficiencies and restricted dietary diversity, and overnutrition, through increased reliance on low-cost, energy-dense, nutrient-poor foods that promote excessive weight gain, contributing to the global double burden of malnutrition [29,30]. In the MENA region, including Lebanon, food insecurity is emerging as an escalating concern, with recent data revealing widespread prevalence among households with children, driven by economic instability, inflation, and declining purchasing power [26].

In this context, FL and NL may act as protective factors, equipping both adolescents and parents with the skills, knowledge, and critical awareness needed to navigate resource constraints while preserving diet quality [17]. Adequate nutrition literacy enhances food skills, self-efficacy for healthy eating, and the adoption of lifelong healthy dietary behaviors among adolescents. Conversely, limited nutrition literacy could exacerbate the adverse effects of food insecurity by decreasing diet quality and increasing susceptibility to stunting [31]. These challenges take on even greater significance in Lebanon, where overlapping crises have magnified food insecurity and nutritional vulnerabilities among youth [32].

### 1.3. The Present Study

In Lebanon, the urgency of this issue is compounded by a prolonged socioeconomic crisis characterized by currency collapse, hyperinflation, political instability, and the deterioration of essential public services [33,34]. The COVID-19 pandemic and the devastating 2020 Beirut port explosion further exacerbated these challenges, deepening social inequalities and severely constraining household incomes [35,36,37]. Poverty rates have escalated, access to affordable nutritious food has declined sharply, and families are increasingly forced to adopt coping mechanisms that threaten dietary adequacy and health [33]. Although national and regional data reveal high rates of food insecurity in households with children [32], evidence remains scarce about its specific implications for adolescent malnutrition, whether undernutrition or overnutrition, when examined in conjunction with both adolescent and parental food and nutrition literacy [26].

Accordingly, this study aims to address this gap by examining the association between adolescents’ nutrition literacy and parental food literacy with both household and adolescent food insecurity, and by exploring how these factors relate to stunting as well as overweight and obesity among Lebanese adolescents. To our knowledge, this is the first study to examine these relationships within both adolescent and parental groups in the Lebanese context.

## 2. Materials and Methods

### 2.1. Human Ethics and Consent to Participate Declaration

The study was conducted in accordance with the ethical principles outlined in the Declaration of Helsinki (2013 revision). Ethical approval was granted by the Ethics Committee of Al-Zahraa University Medical Center, Beirut, Lebanon (Reference 12-2022). Prior to participation, all adolescents and their parents/caregivers were presented with a detailed explanation outlining the study objectives and the voluntary nature of participation. Written informed consent was obtained from all participants before data collection.

### 2.2. Study Design and Participants

A cross-sectional survey was conducted between March and July 2022, targeting Lebanese adolescents and one of their parents or primary caregivers. A representative sample of parent–adolescent dyads was recruited using a stratified cluster sampling approach. The eight Lebanese governorates (Mount Lebanon, Beirut, South Lebanon, North Lebanon, Akkar, Beqaa, Baalbeck-Hermel, and Nabatieh) were used as strata, and within each governorate, households were selected using probability proportional to size sampling. This strategy ensured balanced regional representation and broad geographic coverage.

#### 2.2.1. Sample Size Determination

The required sample size was calculated using the single population proportion formula: *n* = [p(1 − p)(Zα/2)^2^]/e^2^ where *n* is the sample size, Zα/2 = 1.96 at 95% confidence, *p* = 0.05, and *e* = 0.05 (margin of error) [14]. The minimum required sample was 400 dyads. After accounting for an estimated 10% non-response rate, the final target was set at 442 dyads, which was achieved. This number was deemed sufficient based on national statistics (Central Administration of Statistics, 2022; http://www.cas.gov.lb) [38].

#### 2.2.2. Recruitment and Data Collection

Households were approached consecutively within each cluster until quotas were reached. To minimize selection bias, refusals were documented and recruitment relied on multiple entry points, including public spaces, health centers, schools, and local community networks. This approach reduced the risk of over-representing particular subgroups and enhanced the socioeconomic diversity of participants.

A total of 442 Lebanese adolescents and one of their parents or primary caregivers participated. Recruitment was initiated through public announcements, healthcare facilities, and social media platforms, but all data were collected through in-person, face-to-face interviews conducted by a trained research team.

#### 2.2.3. Inclusion and Exclusion Criteria

Eligible adolescents were Lebanese nationals aged 10–19 years, healthy, and free from chronic diseases. Only one adolescent per household was included. Exclusion criteria included current use of iron supplements or blood donation within the month prior to assessment. Parents were eligible if they were Lebanese nationals aged 18–64 years. Households were excluded if either participant declined consent or did not complete study procedures.

#### 2.2.4. Quality Assurance

All interviewers received standardized training on study objectives and data collection procedures, including role-playing and pilot testing. Field supervisors reviewed completed questionnaires daily to detect inconsistencies or missing data. To ensure accuracy, 10% of participants were randomly re-contacted to verify responses. Data entry was performed using double-entry verification to minimize transcription errors.

### 2.3. Questionnaire and Variables

The questionnaire consisted of two main parts: the first part gathered sociodemographic information, while the second part included validated scales assessing nutrition literacy, food security, and nutritional status.

### 2.4. Sociodemographic Information

The first section collected information on adolescents’ age, gender, place of residence, primary caregiver, employment status, education level, school type, and whether they had received nutrition education at school. Parents/caregivers provided their age, education level, and employment status. In the Lebanese context, education levels correspond to elementary (1–6 years), intermediate (7–9 years), secondary (10–12 years), and university (>12 years) of formal education [39]. To assess the family’s socioeconomic status, the Household Crowding Index (HCI) was used, consisting of the total number of household members divided by the number of rooms in the dwelling, excluding bathrooms and kitchens [40]. These variables were used as covariates in subsequent analyses to examine their potential influence on food and nutrition literacy and food insecurity outcomes.

### 2.5. Adolescent Nutrition Literacy

The second section began with the assessment of adolescents’ capacity to access, understand, and apply nutrition-related information using the validated Arabic Adolescent Nutrition Literacy Scale (ANLS) [41]. The ANLS comprises 22 items scored on a five-point Likert scale (1 = strongly disagree to 5 = strongly agree), with total scores ranging from 22 to 110. It covers three domains:

- Functional Nutrition Literacy (FNL) (7 items) covering comprehension and use of basic nutrition information.
- Interactive Nutrition Literacy (INL) (6 items) covering communication and information-seeking skills.
- Critical Nutrition Literacy (INL) (9 items) covering ability to evaluate and apply nutrition information critically.

Scores were categorized into low (22–57.2), moderate (57.2–74.8), and high (74.8–110) levels, based on cut-offs used in previous Arabic validation studies. This classification distinguished between adolescents with poor, moderate, or adequate literacy, providing a nuanced understanding of literacy as a determinant of nutritional outcomes [41].

### 2.6. Parental Food Literacy

Parental food literacy was assessed using the validated 12-item Short Food Literacy Questionnaire (SFLQ) [42]. This instrument, originally developed and validated in Switzerland (Cronbach’s α = 0.82) [14]. The scale has been validated in Arabic [43]. Responses were collected on four- or five-point Likert scales, depending on the items, yielding a total score ranging from 7 to 52. Higher scores reflected better food literacy. In line with previous validation, a median cut-off of 31 was applied to categorize parents into two groups: poor food literacy and adequate food literacy [14].

### 2.7. Household and Adolescents’ Food Security

Household-level and adolescents’ self-reported food security were measured using the Arab Family Food Security Scale (AFFSS), a 7-item validated tool adapted for the Lebanese context. Each affirmative response was assigned one point, with a total score range of 0–7. Consistent with established thresholds, security levels were classified as:

- Food secure (0–1);
- Moderately food insecure (2–4);
- Severely food insecure (5–7).

This classification enabled us to capture the spectrum of food insecurity severity in households and to analyze its relationship with both parental literacy and adolescent nutritional status [44,45].

Adolescents’ own food insecurity experiences were assessed using the validated 14-item Child Food Security Scale (CFS), developed in Lebanon through literature review and qualitative research [45,46]. The items span five domains: (1) cognitive awareness of food scarcity (e.g., caretaker unable to buy food, food running out), (2) emotional awareness (e.g., worry about food running out, parents frustrated), (3) physical awareness (e.g., feeling hungry or weak due to lack of food), (4) child-initiated coping strategies (e.g., reducing portion size, skipping meals, eating at relatives’ homes), and (5) shared responsibility for resources (e.g., child working or saving money to support the family). Each item referred to a six-month recall period (“since the end of the last academic year/beginning of summer”) and was rated on a three-point scale (never, sometimes, often). Total scores ranged from 0 to 14, with higher scores reflecting greater severity of food insecurity [45,46].

The detailed response options and distributions of sociodemographic variables, literacy scores, and food security categories are presented in Table 1 (parents/caregivers) and Table 2 (adolescents), which complement the description of the instruments provided above.

### 2.8. Anthropometrics

Anthropometric measurements of adolescents were conducted by a trained research team under standardized conditions. Each measurement was taken three times, and the average value was recorded. Body weight was assessed to the nearest 0.1 kg using an electronic scale (AMBER Body Scale, NUMED SARL, Beirut, Lebanon), with participants wearing minimal clothing and no footwear. Height was measured to the nearest 0.1 cm using a portable stadiometer (Portable Height Scale, NUMED SARL, Beirut, Lebanon). Height-for-age Z-scores (HAZ) and body mass index-for-age Z-scores (BAZ) were calculated using the WHO Anthro-Plus software (version 1.0.4, Department of Nutrition, World Health Organization, Geneva, Switzerland). Adolescents were classified as moderately or severely stunted and thin if their HAZ and BAZ values were below −2 SD and −3 SD, respectively. Conversely, BAZ values above +1 SD and +2 SD were used to define overweight and obesity, respectively [47].

### 2.9. Statistical Analysis

The SPSS software version 27 was used for the statistical analysis. The Chi-2 test/Fisher exact test were used to compare two categorical variables, whereas Student’s *t*-test was used to compare two means. Multivariable regressions were conducted to compare the levels of food insecurity as reported by adolescents and parents two by two. Variables that showed a *p* < 0.250 were considered as independent variables in the final models. Multicollinearity was verified by the absence of correlations higher than 0.8 between two independent variables. *p* < 0.05 was deemed statistically significant. We calculated effect sizes to quantify the magnitude of associations and group differences and provide information on the practical significance of findings. For Chi-square tests, we reported Cramer’s V, which measures the strength of association between categorical variables independent of sample size. For *t*-tests, we reported Cohen’s d, which expresses the standardized mean difference between two groups.

## 3. Results

A higher percentage of mothers resided in Mount Lebanon, were unemployed, and confessed that they have no salary compared to males. Moreover, a higher mean age and lower mean SFLQ scores were found in fathers compared to mothers. The majority of parent/caregiver respondents were female (72.2%), with a mean age of 45.1 ± 7.3 years and an average of 2.40 children; the largest share resided in Mount Lebanon (37.8%). The majority were married (93.0%) and had at least an intermediate education (83.5%). Employment was predominantly unemployed (55.0%), and household food security was distributed as 38.7% food secure, 31.7% moderately food insecure, and 29.6% severely food insecure. Other sociodemographic details are presented in Table 1.

A significantly higher percentage of boys resided in Mount Lebanon, were enrolled in elementary or intermediate school level, and attended public schools compared to girls. Moreover, lower mean age, functional nutrition literacy and interactive nutrition literacy scores were found in boys compared to girls. Adolescents were mostly female (55.7%), with a mean age of 14.67 ± 2.94 years; three-quarters had at least an intermediate education (≈74.6%), and most were not working (93.4%), with half attending private schools (50.2%). Nutritional status screening showed that 69.5% were neither overweight nor obese, 4.1% had stunting, and 2.7% had wasting. Other sociodemographic details are presented in Table 2.

A higher percentage of girls had significantly adequate FNL and INL scores compared to boys, whereas a higher percentage of mothers had adequate SFLQ scores (Table 3).

A higher percentage of adolescents with severe food insecurity was found in participants having no nutrition education in school curriculum and who had poor FNL scores (Table 4).

### 3.1. Multivariable Analysis

The results of the logistic regression taking the adolescent food insecurity status as the dependent variable, showed that residing in Beirut compared to Mount Lebanon (aOR = 3.20) was significantly associated with higher odds of having moderate food insecurity (Table 5, Model 1).

Having a university level of education compared to elementary (aOR = 0.17), having not received (aOR = 0.15) and having received (aOR = 0.24) nutrition education in school curriculum compared to those not attending school and having higher FNL scores (aOR = 0.43) were significantly associated with lower odds of having severe food insecurity, whereas living in Akkar compared to Mount Lebanon (aOR = 2.92) was significantly associated with higher odds of having severe food insecurity (Table 5, Model 2).

### 3.2. Bivariate Analysis of Factors Associated with the Household Food Insecurity Status

Residing in North Lebanon and Akkar, having an elementary education level, being unemployed, having a monthly salary less than 1.5 million Lebanese Pounds were significantly associated with severe food insecurity. Moreover, higher mean household crowding index and lower SFLQ scores were significantly associated with severe food insecurity (Supplementary Table S1).

### 3.3. Multivariable Analysis

The results of the logistic regression taking household food insecurity status as the dependent variable showed that residing in South Lebanon compared to Mount Lebanon (aOR = 0.25), having a monthly salary of USD 300 compared to no salary (aOR = 0.17), confessing that the salary increased vs. not receiving a salary (aOR = 0.20) were significantly associated with lower odds of moderate food insecurity. Furthermore, a higher HCI (aOR = 1.57) was significantly associated with higher odds of moderate food insecurity (Table 6, Model 1).

Residing in Beirut compared to Mount Lebanon (aOR = 0.16), having a monthly income between USD 100 and 300 (aOR = 0.10) and more than USD 300 (aOR = 0.14) compared to no salary and higher SFLQ scores (aOR = 0.94) were significantly associated with lower odds of having severe food insecurity, whereas a higher HCI (aOR = 1.88) was significantly associated with higher odds of having severe food insecurity (Table 6, Model 2).

### 3.4. Bivariate Analysis of Factors Associated with Overweight/Obesity

A higher percentage of overweight/obese adolescents was found in those attending private and public schools, whereas a higher mean BMI in parents was found in overweight/obese adolescents (Supplementary Table S2).

### 3.5. Multivariable Analysis

Attending private schools vs. not attending school (aOR = 2.97) and a higher BMI in parents (aOR = 1.12) were significantly associated with higher odds of being overweight/obese, whereas a higher HCI (aOR = 0.71) and a higher child food security score (aOR = 0.97) were significantly associated with lower odds of being overweight/obese (Table 7).

## 4. Discussion

This study aimed to examine the associations between adolescent nutrition literacy and parental food literacy with household and adolescent food insecurity, and to explore their relationship with stunting and overweight/obesity among Lebanese adolescents.

### 4.1. Parent Food and Nutrition Literacy and Household Food Insecurity

Our results highlight the critical role of parental food literacy in shaping household food security. Higher parental FL scores were linked to a lower likelihood of household food insecurity. This finding is consistent with Hoteit et al., who reported that poor parental food literacy increased the risk of household food insecurity by almost threefold [14]. Parents with stronger food literacy skills are more likely to make informed purchasing decisions, plan balanced meals, and optimize the use of available resources that could help reduce the effects of economic hardship on food availability and quality [3,48].

Geographical variation was evident, with households in South Lebanon showing lower levels of moderate food insecurity compared to those in Mount Lebanon. This finding may be partially explained by the region’s agricultural activity, which can provide households with direct access to food and reduce dependence on purchased goods. Lower household food insecurity in South Lebanon may also reflect agricultural self-provisioning and informal food-sharing practices that stabilize household food access, even if adolescents’ perceptions of scarcity do not materially differ across regions. Comparable results were noted by Herrera et al., in which higher agricultural output was linked to reduced household food insecurity [49].

Urban residence revealed divergent patterns across levels of analysis. In the household models, residing in Beirut was associated with lower odds of severe food insecurity compared to Mount Lebanon, whereas in the adolescent models, Beirut residence was linked to higher odds of moderate food insecurity. This apparent discrepancy can be understood through two mechanisms. First, household and adolescent food insecurity were measured using different instruments and perspectives, with adolescent self-reports often capturing unique, developmentally specific experiences that do not always align with parental reports [14,45]. Second, urban environments such as Beirut often provide broader coping resources, including informal support, labor opportunities, and remittances, which can mitigate the risk of severe household insecurity while adolescents continue to experience day-to-day compromises in food access [50].

In addition, household income emerged as a strong determinant: families with a stable monthly income were less likely to experience moderate or severe food insecurity. This finding aligns with Loopstra & Tarasuk, who demonstrated that low and unstable incomes are among the strongest predictors of food insecurity across diverse settings, limiting the ability to purchase sufficient, nutritious foods [51]. Conversely, a higher household crowding index was associated with greater food insecurity. This relationship reflects the broader link between socioeconomic disadvantage and inadequate access to food, especially in Lebanon, where it has been shown that both the place of residence and the degree of household crowding can influence adolescents’ own experience of food insecurity [14].

### 4.2. Adolescent Food and Nutrition Literacy and Adolescent Food Insecurity

Our findings highlight that both adolescent and parental literacy were significantly associated with household and child food security status, underscoring the dual role of knowledge and access in shaping nutritional well-being. These results align with previous research from middle- and high-income countries demonstrating that low parental food literacy is linked to poorer child dietary quality and increased risk of food insecurity [41]. Importantly, our results extend this evidence to the Lebanese context, where economic crisis, food price inflation, and structural inequalities exacerbate the consequences of limited literacy.

Compared with studies from Australia, Europe, and the United States, our observed literacy levels were lower, reflecting differences in educational systems, cultural exposure to nutrition information, and the absence of structured national programs promoting food and nutrition literacy in Lebanon [14]. Similar trends were reported in Jordan and Egypt, suggesting that limited literacy may be a regional concern requiring integrated policy action [52].

With respect to nutritional outcomes, adolescents with lower nutrition literacy and higher food insecurity were more likely to be overweight/obese, consistent with international findings linking poor literacy with unhealthy dietary behaviors, including frequent consumption of energy-dense, nutrient-poor foods [53]. This paradox of simultaneous food insecurity and overweight reflects the nutrition transition in Lebanon and highlights the urgent need for double-duty actions.

From a methodological perspective, our recruitment strategy ensured representation across all Lebanese governorates and socioeconomic groups, yet some degree of selection bias cannot be ruled out. For example, households refusing participation may have differed in literacy or food insecurity status. While quality control procedures (e.g., SOPs, inter-rater reliability, 10% re-contact verification) minimized reporting bias, self-reported data remain subject to recall and social desirability bias. Furthermore, while model fit indices (Nagelkerke R^2^, Hosmer–Lemeshow, AIC/BIC) were reported, no significant interaction or moderation effects were observed. The absence of moderation analyses limits our ability to fully capture complex pathways (e.g., whether parental literacy buffers the effect of food insecurity on adolescent outcomes), and this should be explored in future research.

Policy and programmatic implications emerge clearly from our results. First, interventions to enhance food and nutrition literacy should be family-centered, targeting both parents and adolescents simultaneously. Second, integrating nutrition education into school curricula is critical, given that adolescents with prior school-based exposure demonstrated higher literacy levels. Third, strengthening community-based programs that address food insecurity while embedding literacy components could yield synergistic effects, particularly in vulnerable households. Finally, national policies should consider food literacy as a pillar of resilience in times of crisis, alongside food assistance and price stabilization measures.

Moreover, our results demonstrated that adolescents living in Beirut were more likely to experience moderate food insecurity compared to those in Mount Lebanon. This aligns with evidence from Jomaa et al., who reported that a few years ago, nearly half of households in the Greater Beirut area were experiencing moderate to severe food insecurity, where high living costs, unemployment, and income instability can intensify vulnerability [54]. Also, living in Akkar was linked to a higher likelihood of severe FI compared to Mount Lebanon, as FI is concentrated mostly in conflict-affected regions that witness political chaos, financial instabilities and displacement [2].

Educational attainment also emerged as an important factor. Adolescents who had reached university level were less likely to experience severe food insecurity compared to peers with only elementary education. Remaining in formal schooling, regardless of whether the curriculum included nutrition education, appeared to offer a protective effect compared with being out of school entirely. This is consistent with findings from Monroe-Lord et al., who reported that lower education levels were linked to a greater risk of food insecurity [55], and with evidence showing that nutrition education can further strengthen household resilience to food insecurity [56]. Moreover, in adolescence, age and education are closely linked; educational attainment serves as a more meaningful indicator than chronological age, as it reflects both age progression and continuity of schooling directly relevant to food security. This is consistent with the work of Fram et al., who demonstrated that adolescent food insecurity varies by developmental stage, reinforcing the notion that remaining in school can provide a protective buffer against vulnerability [57].

Moreover, adolescents with higher nutrition literacy scores were less likely to experience severe food insecurity. This mirrors findings from Begley et al., who reported that greater food literacy was associated with reduced risk of food insecurity, likely by equipping individuals with the skills and knowledge to maximize available resources and maintain diet quality even under financial constraints [53]. This relationship may also be explained by the way food literacy nurtures self-efficacy and builds confidence in one’s ability to apply food-related skills effectively, which in turn can strengthen both access to and utilization of available foods, even in challenging circumstances [53,58].

### 4.3. Food Insecurity, Food and Nutrition Literacy, and Adolescent Malnutrition

Our results indicated no significant association between household food insecurity and stunting among adolescents after adjustment. This finding is consistent with the review by Patriota et al., who, despite most individual studies reporting a positive link between food insecurity and stunting, found no significant association in the pooled analysis due to high heterogeneity [59]. Additionally, beyond early childhood, links between household food insecurity and linear growth are heterogeneous and often attenuate once socioeconomic and environmental covariates are considered [60]. As highlighted by Aurino et al., factors such as household wealth, parental education, place of residence, household size, and access to adequate water, sanitation, and hygiene can substantially influence growth outcomes, which could potentially mask the direct impact of food insecurity during later childhood and adolescence, helping to explain the absence of a significant association with stunting in our adolescent sample [60].

In contrast, the relationship with overweight/obesity was clearer. Adolescents attending private schools were more likely to be overweight/obese than adolescents not in school, a finding consistent with Farrag et al.’s review in the MENA region, in which higher socioeconomic position and fee-paying schooling often track greater exposure to energy-dense foods and more sedentary environments [11]. Higher parental BMI was also associated with higher adolescent overweight/obesity, reflecting the established role of parental adiposity as a strong predictor of offspring adiposity through shared genetics, home food environments, and lifestyle behaviors [61].

Two household factors appeared protective against excess weight. First, higher child food security was associated with a lower likelihood of overweight or obesity. This aligns with Kral et al., who found that children from food-insecure households had substantially higher odds of obesity, alongside more frequent eating beyond satiety, greater maternal concern about weight, and more restrictive feeding practices [62]. Similar patterns have been documented across diverse socioeconomic contexts [29,63]. Second, a higher HCI was associated with lower odds of overweight/obesity, likely reflecting the role of socioeconomic constraints in shaping diets. In resource-limited households, food choices may shift toward basic staples and away from the energy-dense, processed foods often linked to excess weight [64].

Finally, the observed association between higher adolescent food and nutrition literacy was associated with less severe food insecurity provides a plausible pathway to healthier weight: better planning, purchasing, and preparation skills are linked to improved diet quality under budget constraints, as shown in studies associating greater food literacy with lower food insecurity and better dietary diversity among youth [14,20,65].

### 4.4. Clinical Implications

Our findings underscore that strengthening food and nutrition literacy among both adolescents and their parents is not merely an educational aspiration, but a tangible public health intervention to safeguard against severe food insecurity. As previously reported in countries like LMICs, integrating nutrition literacy into adolescent development programs can improve diet quality and resilience, even under severe resource constraints [53]. Equipping families with the skills to plan balanced meals, optimize limited resources, and make the most of available foods can help preserve diet quality even during periods of economic strain [66]. For Lebanon, where overlapping crises have strained both household budgets and food systems, embedding nutrition literacy into school curricula, particularly within health, biology, and civic education, could provide adolescents with transferable skills to navigate constrained environments. Parent-focused initiatives, delivered through community centers, primary healthcare facilities, and non-governmental organizations, can complement these efforts by strengthening the home food environment and reinforcing adolescents’ skills at household level.

The strong association between stable household income and reduced food insecurity further highlights the importance of parallel policy measures, such as income support, subsidies for healthy foods, and protection of local food systems, to ensure that knowledge is matched with the material means to act on it.

At the same time, the finding that adolescents from higher-income households and private schools were more likely to be overweight or obese serves as a reminder that wealth alone does not guarantee better health outcomes. Greater access to energy-dense foods, more sedentary lifestyles, and high exposure to fast-food environments can offset the benefits of affluence [67]. Interventions must therefore address both ends of the nutrition spectrum, undernutrition and overnutrition, by combining nutrition education with accessible, culturally relevant opportunities for physical activity and healthy eating. By targeting both knowledge and structural determinants, public health strategies can help break the double burden of food insecurity and obesity affecting Lebanese youth.

Finally, Lebanon’s fragile context requires cross-sectoral coordination between ministries of health, education, agriculture, and social affairs, as well as partnerships with non-governmental organizations and international agencies. Such coordinated action can ensure that nutrition literacy initiatives are scaled up equitably, linked to safety nets, and sustained despite economic and political instability. By simultaneously addressing knowledge, structural access, and environmental factors, these strategies can help break the intergenerational cycle of food insecurity and malnutrition affecting Lebanese youth.

### 4.5. Limitations

Despite the valuable insights offered by this study, certain limitations should be considered when interpreting the findings. The cross-sectional design prevents drawing causal relationships between food and nutrition literacy, food insecurity, and nutritional status. Although data collection was conducted through face-to-face interviews, the reliance on self-reported information for dietary and socioeconomic variables may still introduce recall bias or socially desirable responding. Selection bias is also possible, as households willing to participate may differ systematically from those who declined, despite efforts to use stratified cluster sampling and proportional household selection. While interviewer training, field supervision, and data audits were implemented to ensure consistency and accuracy, unmeasured sources of error cannot be entirely excluded. Finally, although our analyses adjusted for several key factors, we acknowledge that not all potential covariates (e.g., age-education overlap, income-food security interdependence) were controlled for, which may partly influence observed associations.

## 5. Conclusions

In conclusion, this study shows that when it comes to food security, what you know, and what your parents know, really does matter. Both adolescent nutrition literacy and parental food literacy function as protective factors against severe food insecurity, with significant implications for adolescent nutritional outcomes. The findings highlight the importance of integrating literacy-based interventions with structural measures that improve access to affordable, healthy diets. Addressing both educational and socioeconomic determinants is essential, particularly in regions facing heightened vulnerability. Future research should adopt longitudinal designs to clarify how literacy and household livelihoods interact over time to influence adolescent health trajectories.

## Figures and Tables

**Table 1:** Demographic and socioeconomic characteristics of parent participants and the household food security status.

Variable	Total	Females	Males	*p*	Effect Size
**Gender**	442	319 (72.2%)	123 (27.8%)		
**Residence**				**0.004**	0.219
Mount Lebanon	167 (37.8%)	127 (39.8%)	40 (32.5%)		
Beirut	27 (6.1%)	24 (7.5%)	3 (2.4%)		
South Lebanon	62 (14%)	43 (13.5%)	19 (15.4%)		
North Lebanon	59 (13.3%)	39 (12.2%)	20 (16.3%)		
Akkar	39 (8.8%)	25 (7.8%)	14 (11.4%)		
Nabatieh	33 (7.5%)	28 (8.8%)	5 (4.1%)		
Beqaa	28 (6.3%)	18 (5.6%)	10 (8.1%)		
Baalbeck-Hermel	27 (6.1%)	15 (4.7%)	12 (9.8%)		
**Marital status**				0.319	0.089
Married	411 (93%)	293 (91.8%)	118 (95.9%)		
Divorced	11 (2.5%)	9 (2.8%)	2 (1.6%)		
Widowed	20 (4.5%)	17 (5.3%)	3 (2.4%)		
**Education level**				0.706	0.070
Illiterate	19 (4.3%)	8 (2.5%)	11 (8.9%)		
Elementary school level	54 (12.2%)	34 (10.7%)	20 (16.3%)		
Intermediate school level	117 (26.5%)	83 (26%)	34 (27.6%)		
Secondary school level	111 (25.1%)	77 (24.1%)	34 (27.6%)		
University level	141 (31.9%)	117 (36.7%)	24 (19.5%)		
**Job status**				**<0.001**	0.526
Unemployed	243 (55%)	225 (70.5%)	18 (14.6%)		
Full-time job	78 (17.6%)	39 (12.2%)	39 (31.7%)		
Part-time job	41 (9.3%)	26 (8.2%)	15 (12.2%)		
Self-employed	80 (18.1%)	29 (9.1%)	51 (41.5%)		
**Monthly income**				0.554	0.105
None	36 (8.1%)	29 (9.1%)	7 (5.7%)		
Less than 1.5 million Lebanese Pounds (LBP)	55 (12.4%)	31 (9.7%)	24 (19.5%)		
LBP 1.5–3 million	129 (29.2%)	87 (27.3%)	42 (34.1%)		
More than LBP 3 million	71 (16.1%)	42 (13.2%)	29 (23.6%)		
Less than USD 100	39 (8.8%)	32 (10%)	7 (5.7%)		
USD 100–300	59 (13.3%)	50 (15.7%)	9 (7.3%)		
More than USD 300	53 (12%)	48 (15%)	5 (4.1%)		
**Impact of economic crisis on income**				**0.046**	0.160
I already have no salary	67 (15.2%)	55 (17.2%)	12 (9.8%)		
I remain with no salary at all	34 (7.7%)	23 (7.2%)	11 (8.9%)		
I earn less than half the salary	47 (10.6%)	39 (12.2%)	8 (6.5%)		
I earn half the salary	54 (12.2%)	42 (13.2%)	12 (9.8%)		
My salary does not change	206 (46.6%)	139 (43.6%)	67 (54.5%)		
My salary increases	34 (7.7%)	21 (6.6%)	13 (10.6%)		
**Household food insecurity status**				0.671	0.043
Food secure	171 (38.7%)	124 (38.9%)	47 (38.2%)		
Mild food insecurity	140 (31.7%)	104 (32.6%)	36 (29.3%)		
Severe food insecurity	131 (29.6%)	91 (28.5%)	40 (32.5%)		
	**Mean ± SD**				
**Age in years**	45.07 ± 7.33	43.40 ± 6.83	49.39 ± 6.82	**<0.001**	0.878
**Household crowding index**	1.28 ± 0.78	1.28 ± 0.82	1.27 ± 0.68	0.887	0.015
**Number of children**	2.40 ± 0.61	2.41 ± 0.61	2.38 ± 0.62	0.697	0.041
**Body Mass Index**	26.21 ± 4.44	26.24 ± 4.55	26.13 ± 4.14	0.826	0.023
**Short Food Literacy Questionnaire total**	31.67 ± 8.49	32.80 ± 7.91	28.76 ± 9.26	**<0.001**	0.486

Numbers in bold indicate significant *p* values.

**Table 2:** Adolescents’ demographic characteristics and nutrition status, and the self-reported adolescent food security status.

Variable	Total	Females	Males	*p*	Effect Size
**Gender**	442	246 (55.7%)	196 (44.3%)		
**Residence**				**0.004**	0.219
Mount Lebanon	167 (37.8%)	82 (33.3%)	85 (43.4%)		
Beirut	25 (5.7%)	19 (7.7%)	6 (3.1%)		
South Lebanon	60 (13.6%)	40 (16.3%)	20 (10.2%)		
North Lebanon	59 (13.3%)	40 (16.3%)	19 (9.7%)		
Akkar	39 (8.8%)	24 (9.8%)	15 (7.7%)		
Nabatieh	35 (7.9%)	15 (6.1%)	20 (10.2%)		
Beqaa	31 (7%)	17 (6.9%)	14 (7.1%)		
Baalbeck-Hermel	26 (5.9%)	9 (3.7%)	17 (8.7%)		
**Education level**				**<0.001**	0.261
Elementary school level	112 (25.3%)	51 (20.7%)	61 (31.1%)		
Intermediate school level	119 (26.9%)	52 (21.1%)	67 (34.2%)		
Secondary school level	122 (27.6%)	74 (30.1%)	48 (24.5%)		
University level	89 (20.1%)	69 (28.0%)	20 (10.2%)		
**School type**				**0.002**	0.169
I am currently not attending school	51 (11.5%)	40 (16.3%)	11 (5.6%)		
Public school	168 (38%)	85 (34.6%)	83 (42.6%)		
Private school	222 (50.2%)	121 (49.2%)	101 (51.8%)		
**Primary caregiver**				0.629	0.076
None	2 (0.5%)	1 (0.4%)	1 (0.5%)		
Mom	15 (3.4%)	8 (3.3%)	7 (3.6%)		
Dad	8 (1.8%)	4 (1.6%)	4 (2.0%)		
Both	407 (92.1%)	225 (91.5%)	182 (92.9%)		
Others	10 (2.3%)	8 (3.3%)	2 (1.0%)		
**Working status**				0.225	0.058
No	413 (93.4%)	233 (94.7%)	180 (91.8%)		
Yes	29 (6.6%)	13 (5.3%)	16 (8.2%)		
**Nutrition education in school curriculum**					
I am currently not attending school	135 (30.5%)				
No	274 (62%)				
Yes	33 (7.5%)				
**Stunting**				0.337	0.046
No	424 (95.9%)	234 (95.1%)	190 (96.9%)		
Yes	18 (4.1%)	12 (4.9%)	6 (3.1%)		
**Wasting**				0.115	0.075
No	430 (97.3%)	242 (98.4%)	188 (95.9%)		
Yes	12 (2.7%)	4 (1.6%)	8 (4.1%)		
**Overweight/Obesity**				0.091	0.080
No	307 (69.5%)	179 (72.8%)	128 (65.3%)		
Yes	135 (30.5%)	67 (27.2%)	68 (34.7%)		
**Household food security status**				0.917	0.020
Food secure	144 (32.6%)	82 (33.3%)	62 (31.6%)		
Mild food insecurity	115 (26%)	64 (26.0%)	51 (26.0%)		
Severe food insecurity	183 (41.4%)	100 (40.7%)	83 (42.3%)		
	**Mean ± SD**				
**Age in years (10–18)**	14.67 ± 2.94	15.31 ± 2.96	13.86 ± 2.72	**<0.001**	0.505
**Functional nutrition literacy total**	22.43 ± 5.86	23.22 ± 5.64	21.45 ± 6.00	**0.002**	0.304
**Interactive nutrition literacy total**	17.70 ± 18	18.20 ± 4.88	17.07 ± 5.45	**0.024**	0.220
**Critical nutrition literacy total**	30.10 ± 7.10	30.41 ± 6.92	29.72 ± 7.33	0.317	0.096
**Child food security**	8.46 ± 8.62	8.29 ± 8.33	8.67 ± 8.99	0.642	0.045

Numbers in bold indicate significant *p* values.

**Table 3:** The status of adolescents’ nutrition literacy and parental food literacy.

Variable	Females	Males	*p*	Effect Size
**Functional nutrition literacy score**			**0.001**	0.159
Poor	89 (36.2%)	102 (52.0%)		
Adequate	157 (63.8%)	94 (48.0%)		
**Interactive nutrition literacy score**			**0.021**	0.110
Poor	96 (39.0%)	98 (50.0%)		
Adequate	150 (61.0%)	98 (50.0%)		
**Critical nutrition literacy score**			0.223	0.058
Poor	100 (40.7%)	91 (46.4%)		
Adequate	146 (59.3%)	105 (53.6%)		
**Child food security score**			1	0.001
Poor	123 (50.0%)	98 (50.0%)		
Adequate	123 (50.0%)	98 (50.0%)		
**Short Food Literacy Questionnaire score**			**<0.001**	0.161
Poor	132 (41.4%)	73 (59.3%)		
Adequate	187 (58.6%)	50 (40.7%)		

Numbers in bold indicate significant *p* values.

**Table 4:** The contribution of the adolescents’ nutrition literacy and parental food literacy to predicting the household and adolescents’ self-reported food insecurity and adolescents’ nutrition status.

Variable	Food Security Status			*p*	Effect Size
	Food Secure 144 (32.6%)	Mild Food Insecure 115 (26%)	Severe Food Insecure 183 (41.4%)		
**Age**	14.89 ± 2.85	14.89 ± 3.09	14.36 ± 2.91	0.175	0.008
**Functional nutrition literacy score**	23.01 ± 5.38	22.85 ± 5.79	21.24 ± 6.38	**0.020**	0.018
**Interactive nutrition literacy score**	18.06 ± 4.93	17.84 ± 5.25	17.08 ± 5.36	0.246	0.006
**Critical nutrition literacy score**	29.78 ± 6.90	30.45 ± 6.53	30.15 ± 7.95	0.711	0.002
**Gender**				0.917	0.020
Females	82 (56.9%)	64 (55.7%)	100 (54.6%)		
Males	62 (43.1%)	51 (44.3%)	83(45.4%)		
**Education level**				0.154	0.103
Elementary school level	29 (20.1%)	28 (24.3%)	55 (30.1%)		
Intermediate school level	40 (27.8%)	25 (21.7%)	54 (29.5%)		
Secondary school level	42 (29.2%)	39 (33.9%)	41 (22.4%)		
University level	33 (22.9%)	23 (20%)	33 (18%)		
**School type**				0.300	0.074
I am currently not attending school	21 (14.6%)	16 (14%)	14 (7.7%)		
Public school	52 (36.1%)	44 (38.6%)	72 (39.3%)		
Private school	71 (49.3%)	54 (47.4%)	97 (53%)		
**Residence**				0.095	0.155
Mount Lebanon	64 (44.4%)	43 (37.4%)	60 (32.8%)		
Beirut	6 (4.2%)	12 (10.4%)	7 (3.8%)		
South Lebanon	22 (15.3%)	17 (14.8%)	21 (11.5%)		
North Lebanon	17 (11.8%)	12 (10.4%)	30 (16.4%)		
Akkar	7 (4.9%)	12 (10.4%)	20 (10.9%)		
Nabatieh	10 (6.9%)	5 (4.3%)	20 (10.9%)		
Beqaa	9 (6.3%)	9 (7.8%)	13 (7.1%)		
Baalbeck-Hermel	9 (6.3%)	5 (4.3%)	12 (6.6%)		
**Working status**				0.737	0.037
No	133 (92.4%)	109 (94.8%)	171 (93.4%)		
Yes	11 (7.6%)	6 (5.2%)	12 (6.6%)		
**Nutrition education in school** **curriculum**				**0.013**	0.120
I am currently not attending school	35 (24.3%)	28 (24.3%)	72 (39.3%)		
No	99 (68.8%)	79 (68.7%)	96 (52.5%)		
Yes	10 (6.9%)	8 (7%)	15 (8.2%)		
**Stunting**				0.433	0.062
No	136 (94.4%)	110 (95.7%)	178 (97.3%)		
Yes	8 (5.6%)	5 (4.3%)	5 (2.7%)		
**Wasting**				0.774	0.034
No	139 (96.5%)	112 (97.4%)	179 (97.8%)		
Yes	5 (3.5%)	3 (2.6%)	4 (2.2%)		
**Overweight/Obesity**					
No	96 (66.7%)	78 (67.8%)	133 (72.7%)	0.457	0.060
Yes	48 (33.3%)	37 (32.2%)	50 (27.3%)		

Numbers in bold indicate significant *p* values.

**Table 5:** Multivariate analyses: Logistic regression taking the adolescent food security status as the dependent variable, using the ENTER method.

	*p*	aOR	95% CI
Model 1: Moderate food insecurity vs. food security * (Nagelkerke R^2^ = 0.063; Hosmer and Lemeshow test: Chi^2^ = 4.48; *df* = 8; *p* = 0.812; AIC = 351.41; BIC = 365.65)			
Education level (Intermediate vs. Elementary *)	0.260	0.64	0.30; 1.39
Education level (Secondary vs. Elementary *)	0.772	0.90	0.44; 1.83
Education level (University vs. Elementary *)	0.179	0.45	0.14; 1.45
Residence (Beirut vs. Mount Lebanon *)	**0.041**	3.20	1.05; 9.75
Residence (South Lebanon vs. Mount Lebanon *)	0.985	1.01	0.47; 2.16
Residence (North Lebanon vs. Mount Lebanon *)	0.947	1.03	0.44; 2.42
Residence (Akkar vs. Mount Lebanon *)	0.124	2.62	0.80; 6.40
Residence (Nabatieh vs. Mount Lebanon *)	0.414	0.61	0.19; 1.98
Residence (Beqaa vs. Mount Lebanon *)	0.391	1.57	0.56; 4.42
Residence (Baalbeck-Hermel vs. Mount Lebanon *)	0.841	0.89	0.27; 2.88
Nutrition education in school curriculum (No vs. I am currently not attending school *)	0.462	0.68	0.25; 1.89
Nutrition education in school curriculum (Yes vs. I am currently not attending school *)	0.486	0.61	0.16; 2.42
Functional nutrition literacy	0.441	0.81	0.45; 1.40
Model 2: Severe food insecurity vs. food security * (Nagelkerke R^2^ = 0.195; Hosmer and Lemeshow test: Chi^2^ = 7.49; *df* = 8; *p* = 0.485; AIC = 405.22; BIC = 420.37)			
Education level (Intermediate vs. Elementary *)	0.459	0.77	0.39; 1.53
Education level (Secondary vs. Elementary *)	0.067	0.53	0.27; 1.05
Education level (University vs. Elementary *)	**<0.001**	0.17	0.06; 0.46
Residence (Beirut vs. Mount Lebanon *)	0.786	1.19	0.35; 4.06
Residence (South Lebanon vs. Mount Lebanon *)	0.826	0.92	0.43; 1.95
Residence (North Lebanon vs. Mount Lebanon *)	0.099	1.86	0.89; 3.91
Residence (Akkar vs. Mount Lebanon *)	**0.031**	2.92	1.10; 7.73
Residence (Nabatieh vs. Mount Lebanon *)	0.453	1.41	0.57; 3.48
Residence (Beqaa vs. Mount Lebanon *)	0.455	1.48	0.53; 4.12
Residence (Baalbeck-Hermel vs. Mount Lebanon *)	0.389	1.55	0.57; 4.20
Nutrition education in school curriculum (No vs. I am currently not attending school *)	**<0.001**	0.15	0.07; 0.34
Nutrition education in school curriculum (Yes vs. I am currently not attending school *)	**0.015**	0.24	0.08; 0.76
Functional nutrition literacy	**<0.001**	0.43	0.26; 0.70

* Reference group. Numbers in bold indicate significant *p* values.

**Table 6:** Multivariate analyses: Logistic regression taking household food insecurity status as the dependent variable, using the ENTER method.

	*p*	aOR	95% CI
Model 1: Moderate food insecurity vs. food security * (Nagelkerke R^2^ = 0.249; Hosmer and Lemeshow test: Chi^2^ = 11.89; *df* = 8; *p* = 0.156; AIC = 377.89; BIC = 407.80)			
Residence (Beirut vs. Mount Lebanon *)	0.393	1.55	0.57; 4.25
Residence (South Lebanon vs. Mount Lebanon *)	**0.002**	0.25	0.11; 0.60
Residence (North Lebanon vs. Mount Lebanon *)	0.562	0.78	0.33; 1.83
Residence (Akkar vs. Mount Lebanon *)	0.810	0.88	0.30; 2.55
Residence (Nabatieh vs. Mount Lebanon *)	0.442	0.66	0.23; 1.89
Residence (Beqaa vs. Mount Lebanon *)	0.468	1.55	0.47; 5.09
Residence (Baalbeck-Hermel vs. Mount Lebanon *)	0.452	0.66	0.22; 1.97
Education level (Intermediate vs. Elementary *)	0.847	0.91	0.36; 2.33
Education level (Secondary vs. Elementary *)	0.292	1.67	0.64; 4.36
Education level (University vs. Elementary *)	0.139	2.10	0.79; 5.62
Job status (Full-time job vs. unemployed *)	0.968	1.02	0.49; 2.12
Job status (Part-time job vs. unemployed *)	0.623	1.29	0.46; 3.61
Job status (Self-employed vs. unemployed *)	0.939	0.97	0.49; 1.93
Monthly income (Less than LBP 1.5 million vs. None *)	0.615	0.70	0.17; 2.85
Monthly income (LBP 1.5–3 million vs. None *)	0.833	0.88	0.27; 2.91
Monthly income (More than LBP 3 million vs. None *)	0.217	0.46	0.13; 1.58
Monthly income (Less than USD 100 vs. None *)	0.390	0.55	0.14; 2.14
Monthly income (USD 100–300 vs. None *)	0.085	0.32	0.09; 1.17
Monthly income (More than USD 300 vs. None *)	**0.010**	0.17	0.05; 0.66
Impact of economic crisis on income (I remain with no salary at all vs. I already have no salary *)	0.644	0.76	0.23; 2.47
Impact of economic crisis on income (I earn less than half the salary vs. I already have no salary *)	0.128	2.41	0.78; 7.46
Impact of economic crisis on income (I earn half the salary vs. I already have no salary *)	0.305	0.59	0.22; 1.61
Impact of economic crisis on income (My salary does not change vs. I already have no salary *)	0.345	0.68	0.31; 1.52
Impact of economic crisis on income (My salary increases vs. I already have no salary *)	**0.014**	0.20	0.06; 0.73
Household crowding index	**0.046**	1.57	1.01; 2.46
Body mass index in parents	0.501	1.02	0.96; 1.09
Short Food Literacy Questionnaire	0.412	0.99	0.95; 1.02
Model 2: Severe food insecurity vs. food security * (Nagelkerke R^2^ = 0.477; Hosmer and Lemeshow test: Chi^2^ = 8.86; *df* = 8; *p* = 0.354; AIC = 295.51; BIC = 325.19)			
Residence (Beirut vs. Mount Lebanon *)	**0.030**	0.16	0.03; 0.84
Residence (South Lebanon vs. Mount Lebanon *)	0.362	0.65	0.26; 1.64
Residence (North Lebanon vs. Mount Lebanon *)	0.503	0.73	0.28; 1.86
Residence (Akkar vs. Mount Lebanon *)	0.994	1.00	0.33; 3.05
Residence (Nabatieh vs. Mount Lebanon *)	0.598	0.73	0.22; 2.37
Residence (Beqaa vs. Mount Lebanon *)	0.324	2.23	0.45; 11.01
Residence (Baalbeck-Hermel vs. Mount Lebanon *)	0.387	0.57	0.16; 2.02
Education level (Intermediate vs. Elementary *)	0.212	0.540	0.21; 1.42
Education level (Secondary vs. Elementary *)	0.380	0.62	0.22; 1.80
Education level (University vs. Elementary *)	0.762	0.85	0.29; 2.49
Job status (Full-time job vs. unemployed *)	0.491	0.72	0.29; 1.82
Job status (Part-time job vs. unemployed *)	0.465	1.52	0.49; 4.68
Job status (Self-employed vs. unemployed *)	0.113	0.48	0.19; 1.19
Monthly income (Less than LBP 1.5 million vs. None *)	0.209	2.56	0.59; 11.12
Monthly income (LBP 1.5–3 million vs. None *)	0.389	1.783	0.48; 6.64
Monthly income (More than LBP 3 million vs. None *)	0.094	0.28	0.06; 1.24
Monthly income (Less than USD 100 vs. None *)	0.992	0.99	0.24; 4.12
Monthly income (USD 100–300 vs. None *)	**0.011**	0.10	0.02; 0.60
Monthly income (More than USD 300 vs. None *)	**0.020**	0.14	0.03; 0.74
Impact of economic crisis on income (I remain with no salary at all vs. I already have no salary *)	0.368	1.79	0.50; 6.40
Impact of economic crisis on income (I earn less than half the salary vs. I already have no salary *)	0.059	4.06	0.95; 17.35
Impact of economic crisis on income (I earn half the salary vs. I already have no salary *)	0.729	0.81	0.24; 2.74
Impact of economic crisis on income (My salary does not change vs. I already have no salary *)	0.344	1.63	0.59; 4.50
Impact of economic crisis on income (My salary increases vs. I already have no salary *)	0.457	0.57	0.13; 2.50
Household crowding index	**0.006**	1.88	1.20; 2.94
Body mass index in parents	0.777	0.99	0.92; 1.06
Short Food Literacy Questionnaire	**0.006**	0.94	0.90; 0.98

* Reference group. Numbers in bold indicate significant *p* values.

**Table 7:** Multivariate analyses: Logistic regression taking Overweight/Obesity (Yes vs. No *) as the dependent variable, using the ENTER method (Nagelkerke R^2^ = 0.132; Hosmer and Lemeshow test: Chi^2^ = 13.68; *df* = 8; *p* = 0.091; AIC = 520.60; BIC = 565.60).

	*p*	aOR	95% CI
Gender (males vs. females *)	0.433	1.20	0.76; 1.89
Education level (Intermediate vs. Elementary *)	0.140	0.55	0.25; 1.22
Education level (Secondary vs. Elementary *)	0.115	0.36	0.10; 1.28
Education level (University vs. Elementary *)	0.198	0.31	0.05; 1.86
School type (Public school vs. I am currently not attending school *)	0.071	2.80	0.91; 8.58
School type (Private school vs. I am currently not attending school *)	**0.048**	2.97	1.01; 8.74
Residence (Beirut vs. Mount Lebanon *)	0.690	1.24	0.43; 3.55
Residence (South Lebanon vs. Mount Lebanon *)	0.743	1.22	0.38; 3.91
Residence (North Lebanon vs. Mount Lebanon *)	0.748	1.21	0.37; 3.93
Residence (Akkar vs. Mount Lebanon *)	0.677	1.30	0.38; 4.48
Residence (Nabatieh vs. Mount Lebanon *)	1	1	0.27; 3.66
Residence (Beqaa vs. Mount Lebanon *)	0.830	0.87	0.23; 3.27
Residence (Baalbeck-Hermel vs. Mount Lebanon *)	0.872	1.12	0.28; 4.43
Nutrition education in school curriculum (No vs. I am currently not attending school *)	0.862	0.94	0.45; 1.94
Nutrition education in school curriculum (Yes vs. I am currently not attending school *)	0.937	0.96	0.34; 2.69
Marital status (Divorced vs. Married *)	0.302	2.08	0.52; 8.31
Marital status (Widowed vs. Married *)	0.181	2	0.72; 5.53
Age	0.204	0.98	0.95; 1.01
Household crowding index	**0.036**	0.71	0.52; 0.98
Number of children	0.577	0.90	0.63; 1.30
Body mass index in parents	**<0.001**	1.12	1.06; 1.18
Child food security	**0.034**	0.97	0.94; 1

* Reference group. Numbers in bold indicate significant *p* values.

## Data Availability

The original contributions presented in this study are included in the article/Supplementary Materials. Further inquiries can be directed to the corresponding authors.

## Supplementary Materials

The following supporting information can be downloaded at: https://www.mdpi.com/article/10.3390/nu17193140/s1, Table S1: Comparison of variables according to the household food insecurity status as reported by the parents; Table S2: Comparison of variables according to the overweight/obesity status as reported by the adolescents.

## Abbreviations

The following abbreviations are used in this manuscript:

ANLS	Adolescent Nutrition Literacy Scale
AFFSS	Arab Family Food Security Scale
FI	Food Insecurity
FNL	Functional Nutrition Literacy
INL	Interactive Nutrition Literacy
CNL	Critical Nutrition Literacy
NL	Nutrition Literacy
FL	Food Literacy
SFLQ	Short Food Literacy Questionnaire
CFS	Child Food Security
HCI	Household Crowding Index
BMI	Body Mass Index
aOR	Adjusted Odds Ratio
CI	Confidence Interval
SD	Standard Deviation
LBP	Lebanese Pounds
WHO	World Health Organization
MENA	Middle East and North Africa
LMICs	Low- and Middle-Income Countries
