# Supplementary material for: Breaking the Cycle of Malnutrition: The Role of Food and Nutrition Literacy in Addressing Food Insecurity Among Lebanese Adolescents
Source: Nutrients. 2025 Sep 30;17(19):3140. doi: 10.3390/nu17193140 (PMC12526276; doi:10.3390/nu17193140)
Supplement: Supplementary file 1 [file nutrients-17-03140-s001.zip › nutrients-3881468-supplementary.pdf]

Supplementary Materials

Table S1: Comparison of variables according to the household food insecurity status as reported by the parents.

Variable	Food insecurity status			*p*	Effect size
	Food secure	Mild food insecure	Severe food insecure		
**Gender**				0.671	0.043
Females	124 (72.5%)	104 (74.3%)	91 (69.5%)		
Males	47 (27.5%)	36 (25.7%)	40 (30.5%)		
**Residence**				**0.003**	0.192
Mount Lebanon	63 (36.8%)	61 (43.6%)	43 (32.8%)		
Beirut	11 (6.4%)	13 (9.3%)	3 (2.3%)		
South Lebanon	38 (22.2%)	10 (7.1%)	14 (10.7%)		
North Lebanon	20 (11.7%)	17 (12.1%)	22 (16.8%)		
Akkar	9 (5.3%)	12 (8.6%)	18 (13.7%)		
Nabatieh	12 (7.0%)	9 (6.4%)	12 (9.2%)		
Beqaa	7 (4.1%)	10 (7.1%)	11 (8.4%)		
Baalbeck-Hermel	11 (6.4%)	8 (5.7%)	8 (6.1%)		
**Marital status**				0.711	0.049
Married	156 (91.2%)	133 (95%)	122 (93.1%)		
Divorced	5 (2.9%)	2 (1.4%)	4 (3.1%)		
Widowed	10 (5.8%)	5 (3.6%)	5 (3.8%)		
**Education level**				**<0.001**	0.178
Elementary school level	21 (12.3%)	17 (12.1%)	35 (26.7%)		
Intermediate school level	45 (26.3%)	30 (21.4%)	42 (32.1%)		
Secondary school level	39 (22.8%)	41 (29.3%)	31 (23.7%)		
University level	66 (38.6%)	52 (37.1%)	23 (17.6%)		
**Job status**				**0.044**	0.121
Unemployed	88 (51.5%)	73 (52.1%)	82 (62.6%)		
Full-time job	36 (21.1%)	24 (17.1%)	18 (13.7%)		
Part-time job	12 (7.0%)	12 (8.6%)	17 (13.0%)		
Self-employed	35 (20.5%)	31 (22.1%)	14 (10.7%)		
**Monthly income**				**<0.001**	0.321
None	9 (5.3%)	14 (10%)	13 (9.9%)		
Less than 1.5 million Lebanese Pounds (LBP)	11 (6.4%)	12 (8.6%)	32 (24.4%)		
1.5-3 million LBP	30 (17.5%)	44 (31.4%)	55 (42%)		
More than 3 million LBP	36 (21.1%)	25 (17.9%)	10 (7.6%)		
Less than 100 USD	12 (7.0%)	14 (10.0%)	13 (9.9%)		
100-300 USD	36 (21.1%)	19 (13.6%)	4 (3.1%)		
More than 300 USD	37 (21.6%)	12 (8.6%)	4 (3.1%)		
**Impact of economic crisis on income**				0.079	0.138
I already have no salary	24 (14.0%)	25 (17.9%)	18 (13.7%)		
I remain with no salary at all	10 (5.8%)	11 (7.9%)	13 (9.9%)		
I earn less than half the salary	12 (7.0%)	22 (15.7%)	13 (9.9%)		
I earn half the salary	23 (13.5%)	15 (10.7%)	16 (12.2%)		
My salary does not change	81 (47.4%)	61 (43.6%)	64 (48.9%)		
My salary increases	21 (12.3%)	6 (4.3%)	7 (5.3%)		
	**Mean ± SD**				
**Age in years**	45.36 ± 8.03	45.14 ± 6.84	44.60 ± 6.89	0.669	0.002
**Household crowding index**	1.07 ± 0.54	1.26 ± 0.70	1.56 ± 1.02	**<0.001**	0.066
**Number of children**	2.39 ± 0.65	2.43 ± 0.59	2.39 ± 0.59	0.806	0.001
**Body mass index**	25.70 ± 3.90	26.54 ± 4.35	26.50 ± 5.10	0.171	0.008
**Short Food Literacy Questionnaire**	33.76 ± 8.13	32.07 ± 8.22	28.53 ± 8.36	**<0.001**	0.065

Numbers in bold indicate significant p values.

Table S2: Comparison of variables according to the overweight/obesity status as reported by the adolescents.				
**Variable**	**No**	**Yes**	***p***	**Effect size**
**Gender**			0.091	0.080
Females	179 (58.3%)	67 (49.6%)		
Males	128 (41.7%)	68 (50.4%)		
**Residence**			0.868	0.085
Mount Lebanon	19 (6.2%)	6 (4.4%)		
Beirut	112 (36.5%)	55 (40.7%)		
South Lebanon	40 (13%)	20 (14.8%)		
North Lebanon	43 (14%)	16 (11.9%)		
Akkar	25 (8.1%)	14 (10.4%)		
Nabatieh	25 (8.1%)	10 (7.4%)		
Beqaa	24 (7.8%)	7 (5.2%)		
Baalbeck-Hermel	19 (6.2%)	7 (5.2%)		
**Education level**			0.133	0.113
Elementary school level	70 (22.8%)	42 (31.1%)		
Intermediate school level	81 (26.4%)	38 (28.1%)		
Secondary school level	87 (28.3%)	35 (25.9%)		
University level	69 (22.5%)	20 (14.8%)		
**School type**			**0.047**	0.118
I am currently not attending school	43 (14.1%)	8 (5.9%)		
Public school	112 (36.6%)	56 (41.5%)		
Private school	151 (49.3%)	71 (52.6%)		
**Working status**			0.233	0.057
No	284 (93.5%)	129 (95.6%)		
Yes	23 (7.5%)	6 (4.4%)		
**Nutrition education in school curriculum**			0.110	0.100
I am currently not attending school	103 (33.6%)	32 (23.7%)		
No	183 (59.6%)	91 (67.4%)		
Yes	21 (6.8%)	12 (8.9%)		
**Household food security status**			0.457	0.060
Food secure	96 (31.3%)	48 (35.6%)		
Mild food insecurity	78 (25.4%)	37 (27.4%)		
Severe food insecurity	133 (43.3%)	50 (37%)		
**Comparison of variables reported by the parents.**				
**Gender**			1	0.006
Females	221 (72%)	98 (72.6%)		
Males	86 (28%)	37 (27.4%)		
**Marital status**			0.137	0.095
Married	290 (94.5%)	121 (89.6%)		
Divorced	7 (2.3%)	4 (3%)		
Widowed	10 (3.3%)	10 (7.4%)		
**Education level**			0.749	0.052
Elementary school level	53 (17.3%)	20 (14.8%)		
Intermediate school level	77 (25.1%)	40 (29.6%)		
Secondary school level	77 (25.1%)	34 (25.2%)		
University level	100 (32.6%)	41 (30.4%)		
**Job status**			0.833	0.044
Unemployed	168 (54.7%)	75 (55.6%)		

Full-time job	56 (18.2%)	22 (16.3%)		
Part-time job	30 (9.8%)	11 (8.1%)		
Self-employed	53 (17.3%)	27 (20.0%)		
**Monthly income**				
None	22 (7.2%)	14 (10.4%)	0.331	0.125
Less than 1.5 million Lebanese Pounds (LBP)	38 (12.4%)	17 (12.6%)		
1.5-3 million LBP	97 (31.6%)	32 (23.7%)		
More than 3 million LBP	53 (17.3%)	18 (13.3%)		
Less than 100 USD	23 (7.5%)	16 (11.9%)		
100-300 USD	40 (13%)	19 (14.1%)		
More than 300 USD	34 (11.1%)	19 (141%)		
**Impact of economic crisis on income**			0.476	0.101
I already have no salary	49 (16%)	18 (13.3%)		
I remain with no salary at all	22 (7.2%)	12 (8.9%)		
I earn less than half the salary	31 (10.1%)	16 (11.9%)		
I earn half the salary	32 (10.4%)	22 (16.3%)		
My salary does not change	149 (48.5%)	57 (42.2%)		
My salary increases	24 (7.8%)	10 (7.4%)		
**Household food insecurity status**			0.710	0.039
Food secure	115 (37.5%)	56 (41.5%)		
Mild food insecurity	100 (32.6%)	40 (29.6%)		
Severe food insecurity	92 (30%)	39 (28.9%)		
**Age adolescents in years**	14.83 ± 2.96	14.30 ± 2.88	0.083	0.180
**Functional nutrition literacy**	22.51 ± 5.85	22.26 ± 5.90	0.677	0.043
**Interactive nutrition literacy**	17.70 ± 5.12	17.71 ± 5.31	0.979	0.003
**Critical nutrition literacy**	30.16 ± 7.11	29.98 ± 7.11	0.805	0.026
**Child food security**	8.87 ± 8.88	7.53 ± 7.94	0.134	0.155
**Age parents in years**	45.36 ± 7.51	44.41 ± 6.88	0.211	0.129
**Household crowding index**	1.31 ± 0.79	1.20 ± 0.75	0.164	0.144
**Number of children**	2.43 ± 0.60	2.33 ± 0.65	0.127	0.158
**Body mass index in parents**	25.65 ± 4.04	27.46 ± 5.02	**<0.001**	0.414
**Short Food Literacy Questionnaire total**	31.45 ± 8.40	32.19 ± 8.69	0.402	0.0087

Numbers in bold indicate significant p values.
